# Features of non-activation dendritic state and immune deficiency in blastic plasmacytoid dendritic cell neoplasm (BPDCN)

**DOI:** 10.1038/s41408-019-0262-0

**Published:** 2019-12-06

**Authors:** Hannah C. Beird, Maliha Khan, Feng Wang, Mansour Alfayez, Tianyu Cai, Li Zhao, Joseph Khoury, P. Andrew Futreal, Marina Konopleva, Naveen Pemmaraju

**Affiliations:** 1 Department of Genomic Medicine, The University of Texas MD Anderson Cancer Center, Houston, TX USA; 2 Department of Leukemia, The University of Texas MD Anderson Cancer Center, Houston, TX USA; 3 Department of Pathology, The University of Texas MD Anderson Cancer Center, Houston, TX USA

**Keywords:** Genetics research, Leukaemia

## Abstract

Blastic plasmacytoid dendritic cell neoplasm (BPDCN) is a rare, male-predominant hematologic malignancy with poor outcomes and with just one recently approved agent (tagraxofusp). It is characterized by the abnormal proliferation of precursor plasmacytoid dendritic cells (pDCs) with morphologic and molecular similarities to acute myeloid leukemia (AML) and myelodysplastic syndrome (MDS)/chronic myelomonocytic leukemia (CMML) in its presentation within the bone marrow and peripheral blood. To identify disease-specific molecular features of BPDCN, we profiled the bone marrow, peripheral blood, and serum samples from primary patient samples using an in-house hematologic malignancy panel (“T300” panel), transcriptome microarray, and serum multiplex immunoassays. *TET2* mutations (5/8, 63%) were the most prevalent in our cohort. Using the transcriptome microarray, genes specific to pDCs (*LAMP5, CCDC50*) were more highly expressed in BPDCN than in AML specimens. Finally, the serum cytokine profile analysis showed significantly elevated levels of eosinophil chemoattractants eotaxin and RANTES in BPDCN as compared with AML. Along with the high levels of *PTPRS* and dendritic nature of the tumor cells, these findings suggest a possible pre-inflammatory context of this disease, in which BPDCN features nonactivated pDCs.

## Introduction

Blastic plasmacytoid dendritic cell neoplasm (BPDCN) is a rare, highly clinically aggressive hematologic malignancy thought to arise from proliferation of precursor plasmacytoid dendritic cells (pDC). Disease presentation includes skin lesions and disseminated disease in the bone marrow, peripheral blood, and lymph nodes^1–3^. Normal pDCs generate interferons during chronic viral infection, can present antigens, and have potential roles in reducing autoimmune responses^4^. However, little is known about this autoimmune aspect in the context of BPDCN. BPDCN has predominance in males (incidence of 0.05 versus 0.02 in females per 100,000 population), generally occurs in those older than 60, and short overall survivals (8–14 months median overall survivals) that decline with age^5^. Patients are predisposed to acute leukemia transformation, despite multi-agent chemotherapy^5^. After multiple attempts to reclassify the disease due to its protean manifestations, BPDCN was initially labeled under the umbrella of acute myeloid leukemia (AML) and associated neoplasms by the World Health Organization (WHO) in 2008. Then in 2016, it was re-classified by WHO under its own separate category under myeloid malignancies, highlighting its unique biology and clinical features^6,7^. In the absence of standard treatment, current chemotherapy regimens borrowed from lymphoma or acute leukemia have been the preferred approach treating patients with BPDCN. However, long-term effectiveness of these chemotherapy regimens and response vary greatly. One potential breakthrough in the field was the observation of near-universal expression of IL3Rα (IL3RA/CD123) in BPDCN, which led to the first approved therapy that uses a recombinant human interleukin 3α (IL3A) protein conjugated to diphtheria toxin (SL-401)^8^. Therefore, further characterization of its pathobiology is warranted. BPDCN is currently distinguished from AML using positive markers, such as: CD4, CD56, CD123, and TCL1, TCF-4, and CD303; and negative markers: CD3, CD8, CD13, CD19, CD34, and myeloperoxidase (MPO)^9^.

Similar to myeloid hematologic disorders AML and myelodysplastic syndromes (MDS), BPDCN patients acquire somatic point mutations in *TET2* and *TP53*, *ASXL1*, *IDH2*, *NRAS*, and *NPM1*, with *TET2* truncating mutations being the most prevalent and recurrent genomic alteration reported^9–12^. Also consistent with AML, the somatic missense and truncating mutations in *TET2* are mutually exclusive with co-occurring *DNMT3A* and *IDH1/2* in BPDCN^11^. Yet their differential responses to similar therapeutic regimens in clinical trial testing suggests that there are key underlying etiologies that are yet to be determined. We sought to further understand the pathobiologic differences between AML and BPDCN, with emphasis on molecular and cytokine analyses.

## Materials and methods

### Specimens

Collection of specimens was through a protocol approved by the UT MD Anderson Cancer Center Institutional Review Board that included informed consent for tissues used for research purposes. For DNA and RNA assays, we used specimens with >60% blasts, specimens with <60% blasts for which CD56+ flow sorting was successful. Several specimens had insufficient yields for the assays and could not be used. Two patients had mixed BPDCN/AML diagnoses at the time of specimen collection (BPDCN-1, BPDCN-4). We were able to sort for CD45 low blasts for BPDCN-1, but not the second patient BPDCN-4 due to specimen limitations. AML samples with *TET2* mutations were identified by searching clinical records for physician-ordered gene-panel results. In total, we profiled bone marrow, peripheral blood and serum samples from primary patient samples of BPDCN (*N* = 16) and *TET2*-mutated AML (AML^TET2m^) (*N* = 9) (Table 1).

**Table 1:** Patient demographics, characteristics, and assays performed.

Patient ID	Gender	Age at which the specimen was collected	Collection number	Cytogenetics	Blast count	Therapy at time of specimen	Specimen tissue source (PB/BM)	Gene panel	Transcriptome microarray	Serum profilng
BPDCN-1	Male	47	1		23	None-newDx	BM	Y	Y	
BPDCN-2	Male	86	1	46,XY[3]	(BLASTS 68% BM) 32	None-newDx	PB			Y
BPDCN-3	Male	65	1	46,XY[20]	0 (2 % on BM)	CHOP	PB			Y
BPDCN-4	Female	74	1	46,XX,del(5)(q22q35)[1]	4 (35% in BM)	None-newDx	PB			Y
			2	46,XX,del(5)(q22q35)[2]/45,sl,inv(1)(p36.3q25),del(3)(p12),inv(6)(p21.1q24),del(7)(p13p15),add(12)(p11.2),−13,−17, + mar[2]/45,sdl, + der(?)t(1;?)(q12;?),-mar[15]/46,XX[1]	82	MDM2 inhibitor	BM	Y	Y	
BPDCN-5	Male	50	1	46,XY[19]	0 (0%BM)	FA - Flodarabin 25 mg/m^2^ along with cyclophosphamide 200 mg/m^2^ × 3 days	PB			Y
BPDCN-6	Male	69	1	46,XY[20]	0 (4% on BM)		PB			Y
			2	45,XY,t(1;6)(p21;p36.3),del(5)(q13q33),der(7)t(1;7)(q12;p22),−13[1] 46,XY,t(1;6)(p21;p36.3),del(5)(q13q33),der(7)t(1;7)(q12;p22),del(11)(q13q23),del(12)(p11.2p13),add(15)(q15)[10] 46,XY[19]	60		BM		Y	
BPDCN-7	Male	82	1	46,XY[20]	0 (2% on BM)	None-newDx	PB			Y
BPDCN-8	Male	65	1	46,XY,t(5;12)(q31;p13)[1] 46,XY[19]	0 (1% on BM)	None-newDx	PB			Y
BPDCN-9	Male	76	1	46,XY, + 3,−17[1]/46,XY[19]	0 (1% on BM)	CHOP	PB			Y
BPDCN-10	Male	63	1	46,XY,t(3;9)(q25;q34)[1]/46,XY[19]	5	None-newDx	PB			Y
					33	None-newDx	BM	Y		
		64	2	46,XY,t(1;9)(p34;q32),del(6)(q16q27),der(6)t(3;6)(q26.2;p25),−21,add(21)(p13)[1]/46,XY[20]	36,39	CPX	BM		Y	
BPDCN-11	Male	68	1	46,XY[20]	1 (BM showed 1%)	hyper-CVAD	PB			Y
BPDCN-12	Male	72	1	46,XY, + 1,add(1)(p13),der(1)dup(1)(q21q32)add(1)(q42),−2,−4, + 11,add(12)(p11.2), −13,−21, + 2mar[8]/48,XY, + 1,der(1)dup(1)(q21q32)add(1)(q42),−2,−4, + 11,add(12)(p11.2),−13, + 3mar[1]/46,XY[11]	8 (BM showed 46%)	None-newDx	PB			Y
		73	2	Not done	85	SL-401	BM	Y		
			3	47~48,XY, + 1,add(1)(p13),der(1)dup(1)(q21q32)add(1)(q42),−4, + 11,add(12)(p11.2),−13, + 21, + 2~3mar[cp19]/46,XY[1]	15 (BM showed 81%)	SL-401	PB	Y	Y	
BPDCN-13	Female	71	1	46,XX[20]	2	None-newDx	PB			Y
			1,2	46,XX[20]	14, 8	SL-401 on 11/17. None on 10/21	BM	Y	Y	
BPDCN-14	Male	69	1	46,XY[20]	0 (BM 4%)	None-relapsed	PB			Y
BPDCN-15	Male	86	1	46,XY[3]	32 (BM 68%)	None-newDx	PB	Y		
BPDCN-16	Male	20	1	45,XY,del(12)(p13),−13[7]	71 (BM 71%)	None-newDx	PB	Y		

*BM* bone marrow, *PB* peripheral blood

### Gene panel sequencing

Genomic DNA (gDNA) was extracted from eight peripheral blood and bone marrow samples of seven patients with BPDCN using the Frozen Tissue protocol 389 from the QIAamp DNA Mini kit (Qiagen, Inc., Valencia, CA). Two timepoints were sequenced for BPDCN-12. Sequencing was then performed on a new-generation version of our in-house gene panel composed of genes commonly associated with hematological malignancies^13^ using Illumina HiSeq 2000 (Illumina Inc., San Diego, CA) (Supplemental Table 1). An in-house virtual normal control was used to identify somatic point mutation and copy-number alterations as previously described^13^. Because our virtual common normal could not be gender-matched, we were unable to assess alterations in chrX. MutationMapper (cBioPortal)^14^ was used to compile and visualize *TET2* mutations.

### Transcriptome microarray

RNA extraction was performed using the Cell Suspension/Body Fluid protocol from the QIAamp RNeasy Mini kit (Qiagen Inc., Valencia, CA) with elution in 35 µL of RNase-free water. Six BPDCN samples had sufficient quantity and quality for use on the ThermoFisher ThermoFisher Scientific Clariom^TM^ D Pico Assay, human. Thus, 100 ng of RNA from each BPDCN (*N* = 6) and AML (*N* = 7) bone marrow specimens were submitted for processing on this microarray at The University of Texas Southwestern Genomics and Microarray Core Facility. The results were subsequently analyzed using the Transcriptome Analysis Console (TAC) (ThermoFisher Scientific, Waltham, MA). Differentially expressed genes among diagnoses were defined as those genes having gene-level fold changes of <−2 or >2 with gene-level FDR diagnostic F-test < 0.01. Differenitally enriched pathways were identified using the TAC Wiki pathway tool on genes with fold changes of <−4 or >4. Shared pathways were determined by using Gene Set Variation Analysis^15^ on each sample using the C2 subset of the Molecular Signatures Database (MSigDB) that includes 4762 gene sets^16^. The enrichment scores were summed across all BPDCN and AML samples, respectively. Those pathways with the lowest and highest enrichment score sums (<−1 and >1) were compared between BPDCN and AML to determine overlapping pathways.

### Serum multiplex immunoassay

Available serum from BPDCN (*N* = 13) and AML^TET2m^ (*N* = 8) were profiled in triplicate using the Cytokine/Chemokine/Growth Factor 45-Plex Human ProcartaPlex™ Panel 1 (EPX450-12171-901, ThermoFisher Scientific) with the addition of IL-3 Human ProcartaPlex™ Simplex Kit (Invitrogen™). The protocol was followed with the option of overnight incubation of antibody with beads. The samples were profiled in triplicate. Out of 45 factors profiled, we kept those factors (*N* = 21) for which >70% of samples had the data within the standard curves for further analysis. Wilcoxon Rank-Sum test on mean observed concentrations were used to determine differentially expressed proteins. The Benjamini & Hochberg method was applied to calculate the false discovery rate (FDR).

## Results

**Fig. 1:**
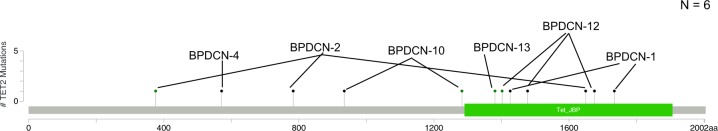
Lollipop plots of *TET2* mutations found in BPDCN patients tested. Annotations are based on NM_001127208.2. The S1674fs and R1476fs mutations in BPDCN-12 were found only in the bone marrow sample that was taken 1 month after the specimen from the peripheral blood, which contained only the R1425X mutation for *TET2*.

Historically, therapies have not been developed specifically for BPDCN due to incomplete knowledge on the underpinnings of the disease. Therefore, we conducted molecular profiling of BPDCN using gene panel sequencing, transcriptome microarray, and serum multiplex immunoassays and cytokine analysis (Table 1). We first assessed somatic point mutations and gene-level copy-number alterations on BPDCN specimens (*N* = 8) using a 300-gene panel that was designed to profile frequently mutated genes in hematologic malignancies and disorders^13^. There, we observed *TET2* mutations in 5/8 (63%) of BPDCN patients, with single or compound truncating and missense mutations scattered throughout the gene (Fig. 1; Supplementary Table 2). Additional mutations were seen in *SRSF2* (recurrent position p.P95L (BPDCN-12) and p.P95R (BPDCN-15)), *STAG2* p.R216X (BPDCN-12), *ASXL1* p.P721fs (BPDCN-4), *EZH2* p.22_22del (BPDCN-12), and *ZRSR2* p.15_18del (BPDCN-10) (Supplementary Table 2). Copy-number alterations were mostly consistent with cytogenetic profiles (Supplementary Table 3). Losses were from three patients (BPDCN-4, BPDCN-10, and BPDCN-12) in chromosomes 3, 5, 7, 9, 12, 13, 17, and 20 (Supplementary Table 3b). Along with the cytogenetics reports, we concluded that our cohort was composed of patients with predominantly *TET2* mutations.

Since *TET2* mutations occur frequently in other myeloid malignancies, these were unlikely to be disease-specific alterations. Therefore, we sought to enhance our ability to observe disease-specific expression signals by comparing BPDCN to AML specimens that had *TET2* mutations. We used available AML^TET2m^ specimens for use in the transcriptome (*N* = 7) and serum (*N* = 8) profiling (Supplementary Table 4). In the transcriptome assay, three of the seven (78%) AML^TET2m^ patients had normal cytogenetics, which is the subset that is reported to have poorer prognosis based on *TET2*-mutated status^17,18^. Healthy control dendritic cells were not readily available for this analysis.

**Fig. 2:**
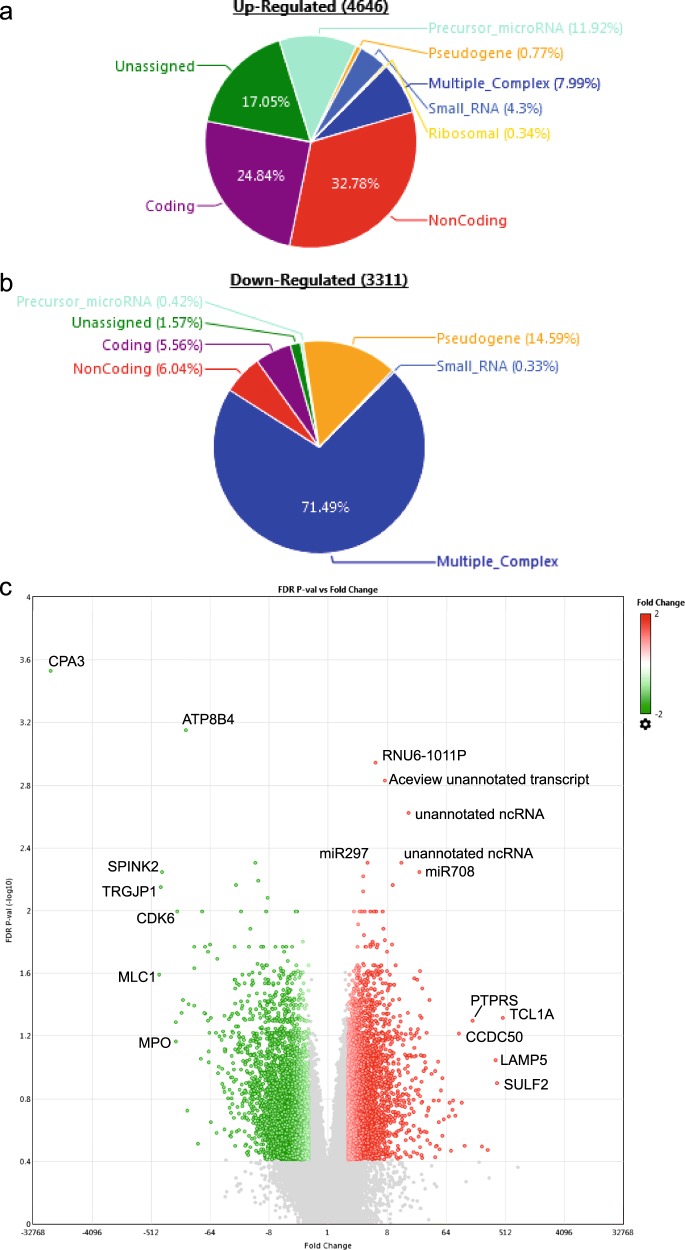
Differential gene expression in BPDCN (*N* = 6) as compared with *TET2*-mutated AML (AML^TET2^) (*N* = 7). Pie charts of the type of transcripts upregulated (**a**) or downregulated (**b**) in BPDCN as compared with AML^TET2^. Coding: transcript results in a protein. Noncoding: transcript does not result in a protein. Pseudogene: transcript may not be expressed and if expressed, does not produce a functional protein. Precursor-microRNA: unprocessed, premature microRNA. Unassigned: uncharacterized transcript. Multiple_Complex: fits into multiple categories of the above. **c** Volcano plot showing the genes that are differentially expressed between BPDCN and AML^TET2^. The genes in red (upregulated in BPDCN) and green (downregulated in BPDCN) are at fold changes −4 > x > 4, with FDR F-test adjusted *P* < 0.03.

Out of the 7957 genes with significantly greater than twofold difference in expression between the BPDCN and AML^TET2m^ samples, 4646 were upregulated and 3311 downregulated in BPDCN as compared with AML^TET2m^ (Fig. 2a–c). Among them, we validated previous findings of higher levels of *TCL1A* and lower levels of *MPO* in BPDCN as compared with AML^TET2m^ (FDR adjusted *P* < 0.05 and *P* < 0.1, respectively) (Fig. 2c; Supplementary Fig. 1a–b, Supplementary Table 5). The 272 significantly differentially expressed pathways included those that were lower in BPDCN than in AML^TET2m^: Cytoplasmic Ribosomal Proteins (Rank: 1), Electron Transport Chain (Rank: 2), Generic Transcription Pathway (Rank: 11). These types of pathways may indicate that BPDCN is more indolent as compared with AML (Supplementary Table 5). None of the most highly enriched pathways (top 400 of 4762 interrogated) in both diseases were shared. Two pathways with the lowest enrichment scores were present in both BPDCN and AML: BIOCARTA_ASBCELL_PATHWAY, the antigen-dependent activation of B cells, and REACTOME_NA_CL_DEPENDENT_NEUROTRANSMITTER. Both of these gene sets had low expression in four of six BPDCN samples, and five of seven AML samples (Supplementary Table 6a, b).

pDC markers *LAMP5*^19^ and *CCDC50*^20^ were higher in BPDCN than in AML^TET2m^ (*P* < 0.01, *P* < 0.001, respectively) (Fig. 2c; Supplementary Fig. 1c, d, Supplementary Table 5). *LAMP5* is expressed in pDCs that are not activated^19^. Thus, the elevated levels here may indicate the state of dendritic nature in BPDCN cells^21^. Given the stimulatory role of NFkB hyperactivation in BPDCN^22^, the upregulated expression of *CCDC50* may negatively regulate NFkB in these patients. From a therapeutic standpoint, this may validate recent efforts to suppress NFkB activation with the proteasome inhibitor bortezomib in order to inhibit cell proliferation, induce cell death, and prolong the survival of BPDCN patients^23^.

**Fig. 3:**
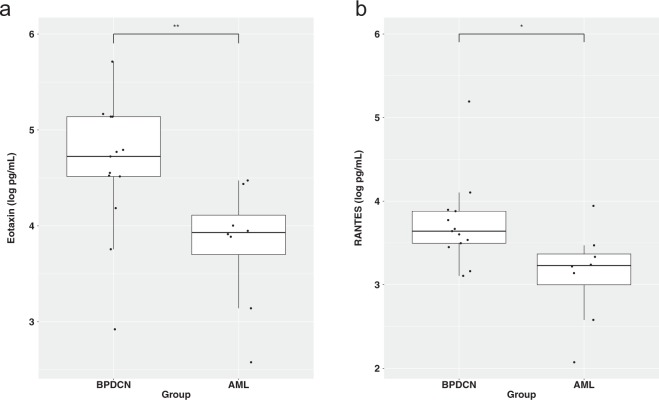
Serum protein levels that were significantly different between BPCN and AML. **a** Eotaxin, **P < 0.01; **b** RANTES, *P < 0.05.

Our data indicated possible links between BPDCN and immune deficiency. *PTPRS* is higher in BPDCN as compared with AML^TET2m^ (FDR adjusted *P* < 0.00001; Supplementary Fig. 1e). Since PTPRS inhibits the activation of nonneoplastic pDCs so as to prevent immune-mediated inflammation^22^, this high expression may be imply that BPDCN cells are derived from nonactivated pDCs in an environment that is poised to become inflammatory. Interestingly, the serum cytokine profiles showed significant higher levels of eotaxin and RANTES in the BPDCN cohort than in the AML^TET2m^ group (Fig. 3a, b; Supplementary Table 7). Both of these are implicated in allergic and autoimmune reactions by behaving as eosinophil chemoattractants. There is little evidence of underlying inflammatory processes since white blood cell counts were low, while there may be an indication for eosinopenia based on absent or low absolute count and proportion of eosinophils (Supplementary Table 8). Therefore, BPDCN may exist in a context in which there is a defect in inflammatory response.

Finally, we observed lower JAK-STAT signaling in BPDCN (Rank:14), which is related to the signaling events that occur downstream of IL-3Rα activation. Lower levels of downstream molecules (*STAT3*, *STAT5B*, *CBL*, and *PTPN6*) and related upstream molecule (*CSF3R*) were notable (Supplementary Figs. 2, 3). In addition, LAMP5 is protein that is relocated to the cell surface of pDCs 24 h after stimulation by IL-3. Its high levels in BPDCN may be inhibiting further IL-3/STAT3 signaling. In contrast, constitutive STAT3 signaling contributes to AML cell proliferation and survival^24,25^.

We also observed several other notable genes: *CPA3*, *ATP8B4*, *SPINK2*, *TRGJP1*, and *CDK6*, which were expressed at lower levels in BPDCN as compared with AML^TET2m^ as well as *SULF2*, miR297, and miR708 that were higher in BPDCN (Fig. 2c). These are potential markers in distinguishing BPDCN from AML, but currently have unclear implications in the etiology of disease.

## Discussion

Previous single-cell sequencing of CD45 + HLA-DR + CD123+ blasts from four BPDCN patients revealed that BPDCN is most closely related to pDCs, with expression of B-cell markers, such as *FCRLA*, *IGLL1*, *TCL1A*, and *IGLL5*^26^. At the same time, patients with BPDCN often share features with patients with AML, including anemia, blasts in the bone marrow, and somatic point mutations in genes such as: *TET2*, *ASXL1*, *IDH2*, and *NPM1*. BPDCN can also progress to AML^5^, has been associated with co-existence of/or prior MDS/CMML in ~20% of patients in one major cohort^27^, and has been detected in a patient with Felty’s syndrome and myelodysplastic syndrome^5,28^. Therefore, BPDCN harbor mixed dendritic and myeloid characteristics. However, the features that distinguish BPDCN from AML have not been explored to great depth. These features are not only potential biomarkers, but could also determine the molecular context for which a dendritic cell that acquires such similar mutations would succumb to a similar etiology as AML. We sought to uncover these types of disease-specific features by conducting transcriptome and serum analyses comparisons of BPDCN and AML samples. We chose to compare BPDCN with *TET2*-mutated AML in order to rule out epigenetic-driven expression patterns that are the result of *TET2* mutations. This group of BPDCN represents the majority of patients, but may exclude other minor subgroups that are yet to be characterized.

In examining the serum, we found elevated levels of eotaxin and RANTES, which suggests that BPDCN patients may be more prone to autoimmunity. Previously, Zhan et al. proposed that susceptibility to autoimmunity could depend on pDC lifespans^29^. This idea was based on the fact that the NZB mouse strain has a longer pDC lifespan than C57BL/6 mice, and is more prone to developing lupus^29^. Transgenic mice that are overexpressing the anti-apoptotic gene *BCL2* have longer-lived pDCs^30^. In BPDCN, *BCL2* levels are higher as compared with normal pDCs^31^, which may indicate that BPDCN cells are comprised long-lived malignant pDCs in patients who are susceptible to autoimmunity. At the same time, BCL2 is advantageous for promoting tumor cell survival, which is consistent with the effectiveness of BCL2 inhibition in BPDCN^32,33^. Moreover, autoimmune pathologies have been hypothesized to damage the bone marrow and induce destruction of myeloid precursor cells^34^. Thus, these links may give insights into the progression of BPDCN to AML. The role of pDC lifespan and state of autoimmunity in these patients should be examined further.

Among the top differentially expressed genes between BPDCN and AML^TET2m^ were specific dendritic markers that we believe are insightful into the dendritic nature of the disease: (*LAMP5*^19^*, CCDC50*^20^). *LAMP5* is within the list of genes that have overlapping expression between healthy pDCs and cDCs by single-cell RNASeq^26^. In addition, the high levels of *LAMP5* is consistent with the immunohistochemical predominance (78%) that was noted in 33 other BPDCN patients^21^.

In its natural context, pDCs serve to recognize foreign particles such as viruses and synthetic oligonucleotides by becoming activated^35^. The canonical activation pathway is through toll-like receptors TLR7/9 that leads to the production of pro-inflammatory cytokines by pDCs in order to regulate activity and to stimulate T lymphocytes^35^. Using both normal pDC and BPDCN cells (CAL-1), Combes et al. demonstrated that initial high levels of LAMP5 (BAD-LAMP) in both of these cell types actively traffic TLR9 to late endosomes as a possible mechanism for preventing cytokine release^36^. BAD-LAMP subsequently decreases upon receiving a CpG activation signal. In our data, no differences in the levels of TLR pathways were seen between BPDCN and AML^TET2m^ at the expression level^36^. This, along with the high levels of *LAMP5*, suggest that the TLR pathways are not differentially activated in BPDCN. Thus, inhibiting this particular pDC activation pathway may be contributing to a state of immune tolerance. Additional support for this are the high levels of *PTPRS* that prevent activation as well as the elevated serum levels of eotaxin and RANTES in BPDCN that would be predicted to elicit an activation response^37^.

By integrating our findings with those of others, we surmise that BPDCN consists of pDCs in a nonactivated survival state within a pre-inflammatory context. BPDCN and AML are distinct, yet related diseases. Understanding the initiation and formation of BPDCN could serve to prevent further progression into AML and also link progenitor cells from the bone marrow to both malignancies and pathogeneses of other autoimmune disorders such as systemic lupus erythematosus (SLE) that have hallmark cutaneous lesions^38^.

## Supplementary information

Supplemental Figures

Supplemental Tables

## Data Availability

Gene panel sequencing and transcriptome microarray data are deposited in the European Genome-phenome Archive under EGAS00001003453.
