# Supplementary material for: Features of non-activation dendritic state and immune deficiency in blastic plasmacytoid dendritic cell neoplasm (BPDCN)
Source: Blood Cancer J. 2019 Dec 6;9(12):99. doi: 10.1038/s41408-019-0262-0 (PMC6898719; doi:10.1038/s41408-019-0262-0)
Supplement: Supplementary file 1 — Supplemental Figures [file 41408_2019_262_MOESM1_ESM.pdf]

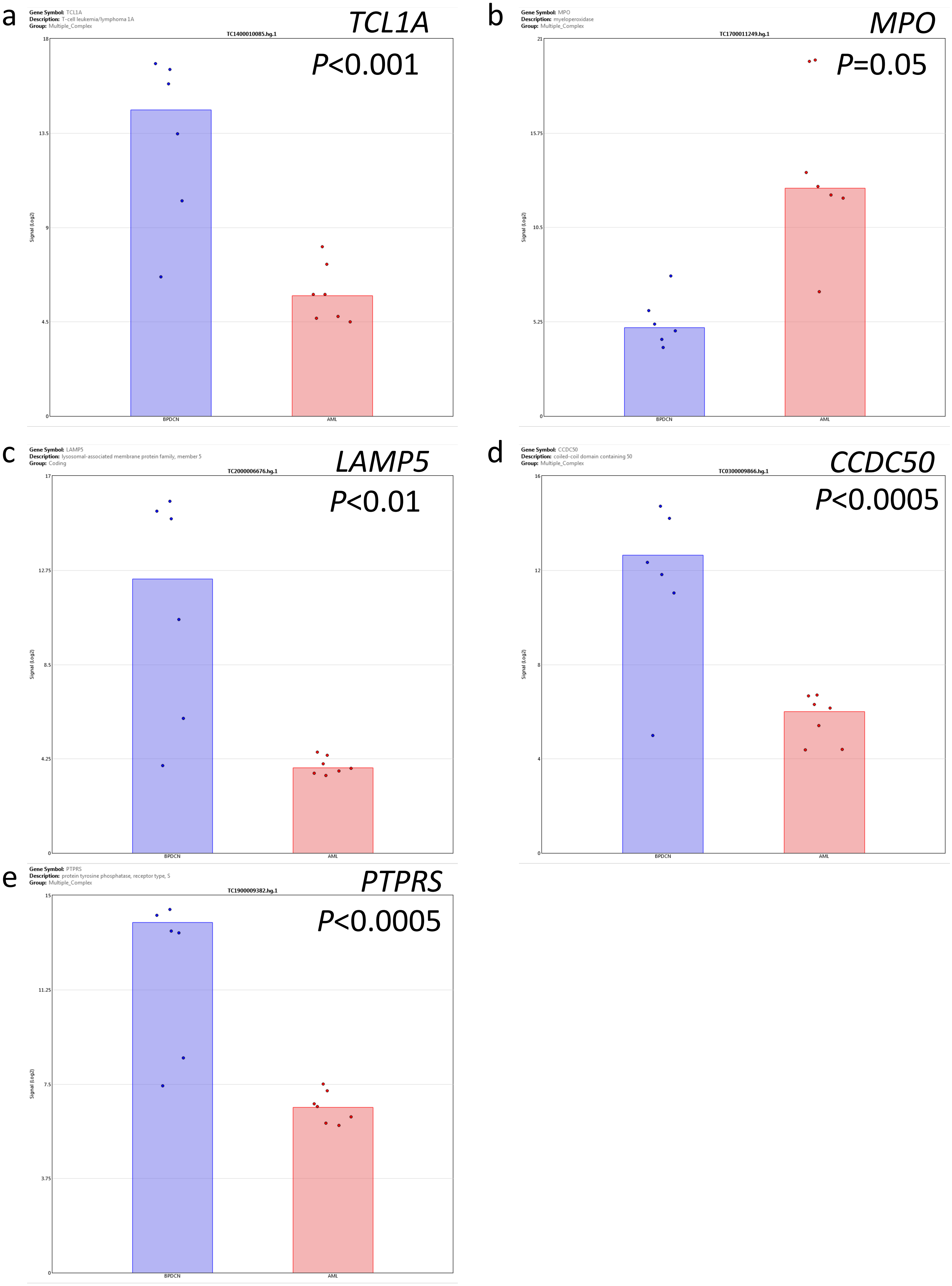
a
Gene Symbol: TCL1A
Description: T-cell leukemia/lymphoma 1A
Group: Multiple_Complex
TC1400010085.hg.1
TCL1A
P<0.001
Signal (Log2)
18
13.5
9
4.5
0
BPDCN
AML
b
Gene Symbol: MPO
Description: myeloperoxidase
Group: Multiple_Complex
TC1700011249.hg.1
MPO
P=0.05
Signal (Log2)
21
15.75
10.5
5.25
0
BPDCN
AML
c
Gene Symbol: LAMP5
Description: lysosomal-associated membrane protein family, member 5
Group: Coding
TC2000006676.hg.1
LAMP5
P<0.01
Signal (Log2)
17
12.75
8.5
4.25
0
BPDCN
AML
d
Gene Symbol: CCDC50
Description: coiled-coil domain containing 50
Group: Multiple_Complex
TC0300009866.hg.1
CCDC50
P<0.0005
Signal (Log2)
16
12
8
4
0
BPDCN
AML
e
Gene Symbol: PTPRS
Description: protein tyrosine phosphatase, receptor type, S
Group: Multiple_Complex
TC1900009382.hg.1
PTPRS
P<0.0005
Signal (Log2)
15
11.25
7.5
3.75
0
BPDCN
AML

Supplemental Figure 1. Differentially expressed genes between BPDCN and AML. (**a**-**b**) Known markers *TCL1A* and *MPO*. (**c-d**) Dendritic cell markers *LAMP5* and *CCDC50* (**e**) *PTPRS*

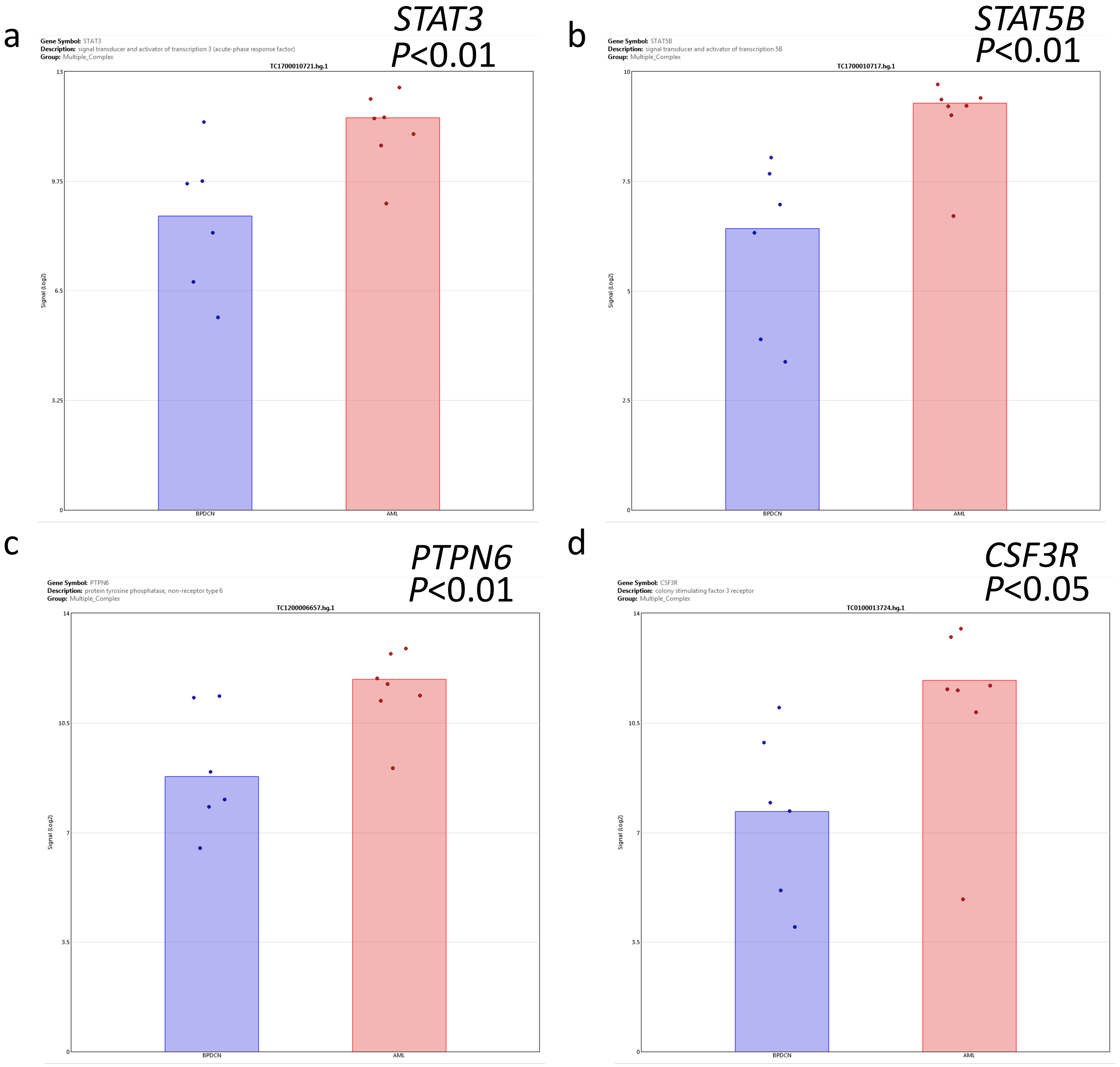
a
STAT3
P<0.01
Gene Symbol: STAT3
Description: signal transducer and activator of transcription 3 (acute-phase response factor)
Group: Multiple_Complex
TC1700010721.hg.1
Signal (Log2)
13
9.75
6.5
3.25
0
BPDCN
AML
b
STAT5B
P<0.01
Gene Symbol: STAT5B
Description: signal transducer and activator of transcription 5B
Group: Multiple_Complex
TC1700010717.hg.1
Signal (Log2)
10
7.5
5
2.5
0
BPDCN
AML
c
PTPN6
P<0.01
Gene Symbol: PTPN6
Description: protein tyrosine phosphatase, non-receptor type 6
Group: Multiple_Complex
TC1200006657.hg.1
Signal (Log2)
14
10.5
7
3.5
0
BPDCN
AML
d
CSF3R
P<0.05
Gene Symbol: CSF3R
Description: colony stimulating factor 3 receptor
Group: Multiple_Complex
TC0100013724.hg.1
Signal (Log2)
14
10.5
7
3.5
0
BPDCN
AML

Supplemental Figure 2. Differentially expressed genes related to IL3 signaling expression (a) *STAT3* (b) *STAT5B* (c) *PTPN6* (d) *CSF3R*

STAT3/5

a

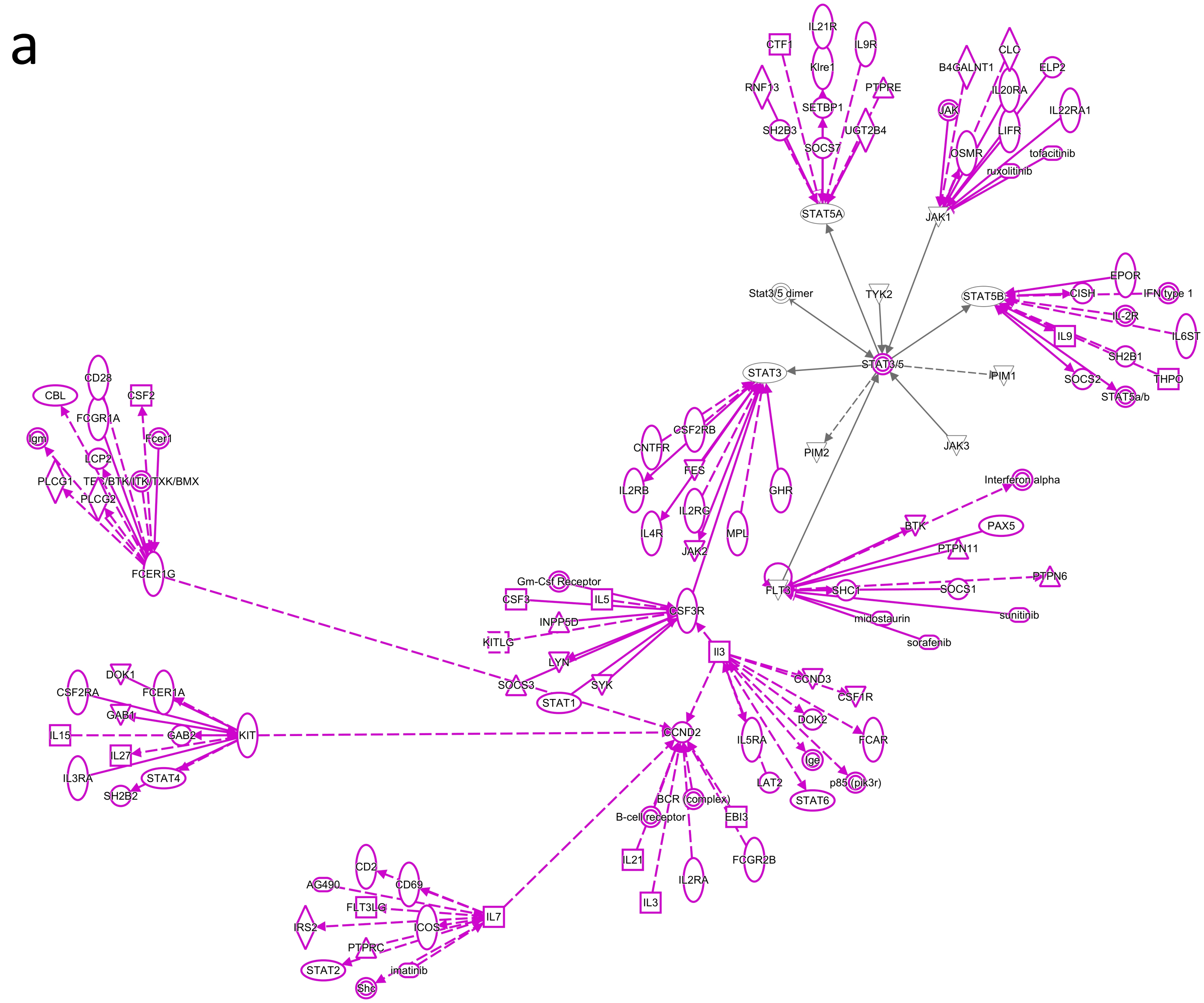

© 2000-2018 QIAGEN. All rights reserved.

b

Consider only molecules and/or relationships where

(cell lines = K-562 OR NB4 OR HMC-1 OR J774 OR J-774A.1 OR SR OR THP-1 OR Lymphoma Cell Lines not otherwise specified OR Jurkat OR HL-60 OR Other Immune cell lines OR U266 OR Other Macrophage Cancer Cell Lines OR RBL-2H3 OR RPMI-8266 OR U937 OR Macrophage Cancer Cell Lines not otherwise specified OR Other Lymphoma Cell Lines OR Other Leukemia Cell Lines OR Leukemia Cell Lines not otherwise specified OR MOLT-4 OR HEL OR Myeloma Cell Lines not otherwise specified OR CCRF-CEM OR Other Myeloma Cell Lines OR BA/F3 OR WEHI-231 OR Immune cell lines not otherwise specified OR RAW 264.7) AND

(diseases = Cancer OR Inflammatory Disease OR Hematological Disease OR Immunological Disease OR Tumor Morphology)

Supplemental Figure 3. Ingenuity Pathway Analysis (IPA) of STAT3 signaling. The networks (a) were generated through the use of IPA (QIAGEN Inc., https://www.qiagenbioinformatics.com/products/ingenuity-pathway-analysis) using the filters shown (b)
